# Supplementary material for: In silico analysis of maize HDACs with an emphasis on their response to biotic and abiotic stresses
Source: PeerJ. 2020 Feb 12;8:e8539. doi: 10.7717/peerj.8539 (PMC7023831; doi:10.7717/peerj.8539)
Supplement: Table S1 [file peerj-08-8539-s001.docx]

**Table S1.** Primers used in this study.

| **Gene ID** | **Primer** | **Sequence (5’ - 3’)** |
| --- | --- | --- |
| GRMZM2G081474 | GRMZM2G081474-F | ACGCTCTTGAGAGTTGAGCA |
|  | GRMZM2G081474-R | CATCGTTTGCAACTGTCGGT |
| GRMZM2G163572 | GRMZM2G163572-F | AGATTCCAAGAGAGCCCACC |
|  | GRMZM2G163572-R | GCACAGCAACTGCCTACTTT |
| GRMZM2G172883 | GRMZM2G172883-F | AAGCAAGCTCCTCAGGCAGA |
|  | GRMZM2G172883-R | CAGGGACGTATTGCGGATTC |
| GRMZM2G136067 | GRMZM2G136067-F | ACACCACCTTTCGTGGTCTG |
|  | GRMZM2G136067-R | CAGCAGCGTGCAACATTTCT |
| GRMZM2G119703 | GRMZM2G119703-F | CCGTCAGGAGTGAAGCGAT |
|  | GRMZM2G119703-R | TCCGGTGCAAGAATTCCACA |
| GRMZM2G367886 | GRMZM2G367886-F | TTACGTCTCCTTCCTCCGGT |
|  | GRMZM2G367886-R | GTATGCAAGGTCACAGCCCT |
| GRMZM2G008425 | GRMZM2G008425-F | AACAGACATCCTCGATGGCG |
|  | GRMZM2G008425-R | CAGCTGGCTGCCTAATCCTA |
| GRMZM2G046824 | GRMZM2G046824-F | CCAAAATCGCCTCATCGACC |
|  | GRMZM2G046824-R | ACGACAGTTCCTGTTCCGAC |
| GRMZM2G056539 | GRMZM2G056539-F | ACAAGCTAGCGGTGTTCTGG |
|  | GRMZM2G056539-R | AGGAGTGGAAGGAGAGGAGC |
| GRMZM2G456473 | GRMZM2G456473-F | AACACTCACCAGGGAGGATG |
|  | GRMZM2G456473-R | ACGAGCATCGAGAAGTGAAT |
| GRMZM2G107309 | GRMZM2G107309-F | CTGCTCCACAGTGAGCTAGAA |
|  | GRMZM2G107309-R | AAGCATGTTCTTTGTCTGCTCG |
| GRMZM2G457889 | GRMZM2G457889-F | AAGGCAAGCAAATGGATGCG |
|  | GRMZM2G457889-R | GCTAGGGCAAGCTCGAATCA |
| GRMZM2G058573 | GRMZM2G058573-F | CAGCTCCAGAGGGTGTCTTG |
|  | GRMZM2G058573-R | TGATATCCCTGCCCCGGTAA |
| GRMZM5G807054 | GRMZM5G807054-F | TGTGGTACATCCATCAGCCG |
|  | GRMZM5G807054-R | CAGGCTTTAGCACTCCACCA |
| GRMZM5G898314 | GRMZM5G898314-F | GGTTCGGAAGAACAGACACCT |
|  | GRMZM5G898314-R | TTGGGGGACTTCTTGTCAGC |
| GRMZM2G100146 | GRMZM2G100146-F | GCCATTGGAACGCTCTCTATC |
|  | GRMZM2G100146-R | TCCTCCTTCCCATCAGCTTTG |
| GRMZM2G159032 | GRMZM2G159032-F | GGAGGAAGACACTCCAACTCC |
|  | GRMZM2G159032-R | GACACCAGATTTTGGCGCAG |
| GRMZM2G057044 | GRMZM2G057044-F  GRMZM2G057044-R | CAGGTGGTGCTGATCCTAGTG  TTGCACTGTTTGCAGAGTGA |
| GRMZM2G126010  (*actin*) | GRMZM2G126010-F | GATGATGCGCCAAGAGCTG |
|  | GRMZM2G126010-R | GCCTCATCACCTACGTAGGCAT |
