# Supplementary figures and images for: In silico analysis of maize HDACs with an emphasis on their response to biotic and abiotic stresses
Source: PeerJ. 2020 Feb 12;8:e8539. doi: 10.7717/peerj.8539 (PMC7023831; doi:10.7717/peerj.8539)

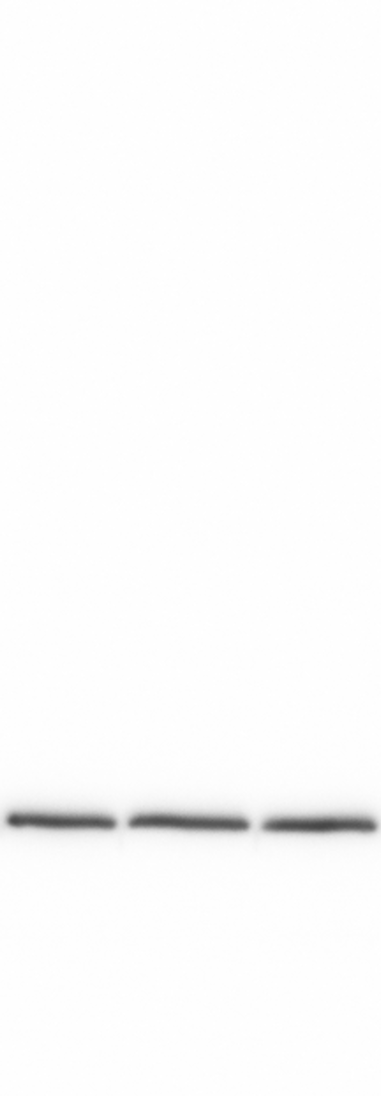

Supplement: Supplemental Information 2 [file peerj-08-8539-s004.zip › H3.tif]

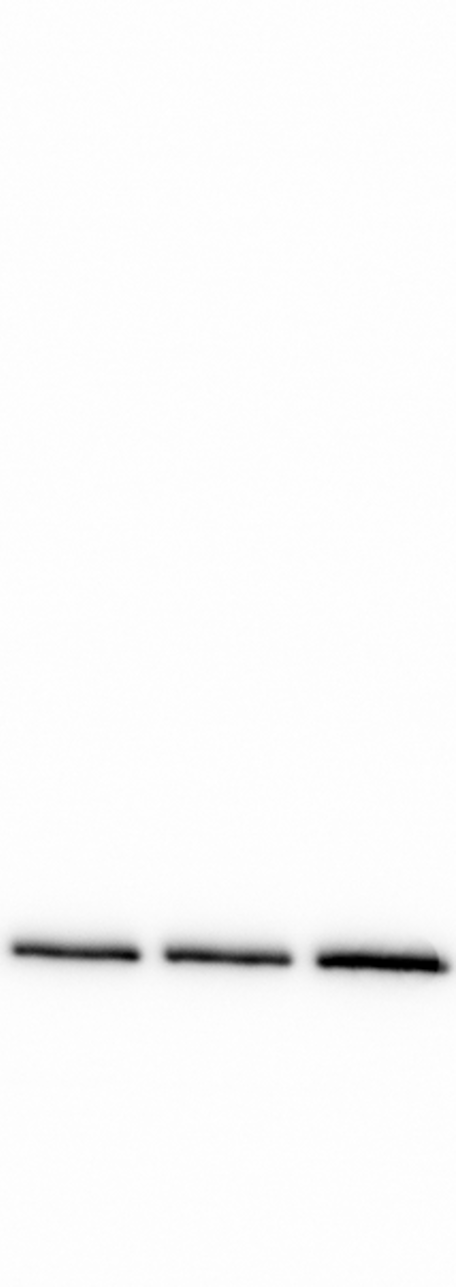

Supplement: Supplemental Information 2 [file peerj-08-8539-s004.zip › H3K9ac.tif]

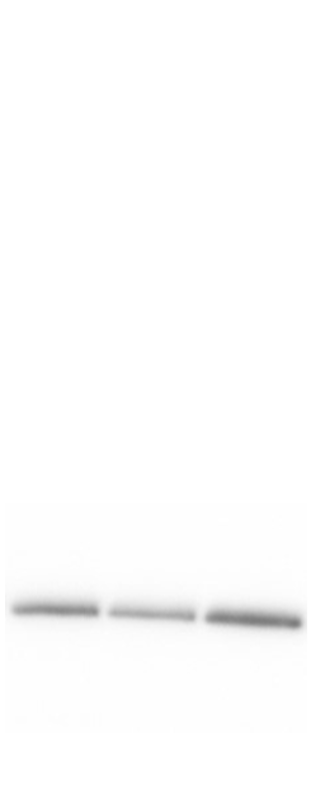

Supplement: Supplemental Information 2 [file peerj-08-8539-s004.zip › H4K5ac.tif]
